# How can counselling by family physicians on nutrition and physical activity be improved: trends from a survey in Germany

**DOI:** 10.1007/s00432-022-04233-5

**Published:** 2022-08-06

**Authors:** S. J. Boesenecker, V. Mathies, J. Buentzel, J. Huebner

**Affiliations:** 1 Clinic for Internal Medicine II, University Hospital, Bachstraße 18, 07743 Jena, Germany; 2 University Tumor Center, University Hospital, Jena, Germany; 3 Clinic for Otorhinolaryngology, Head Neck Surgery, Suedharz Klinikum, Nordhausen, Germany

**Keywords:** Qualitative research, Family practice, Oncology, Access to information, Healthy lifestyle

## Abstract

**Purpose:**

Cancer and its therapy causes severe symptoms, most of which are amendable to nutrition and physical activity (PA). Counselling on nutrition and PA empowers patients to take part more actively in their treatment. Many cancer patients are yet in need of information on these topics. In this study, we investigate the perception of family physicians (FP) on nutrition and PA in cancer patient care and assess barriers and steps to improve their involvement in counselling on these topics.

**Methods:**

Based on qualitative content analysis of 5 semi-structured interviews with FP, a questionnaire was developed and completed by 61 German FP.

**Results:**

Most of the FP acknowledged the importance of nutrition and PA during (91.4%) and after (100%) cancer therapy. While many participants were involved in cancer patient care, 65.6% of FP viewed themselves as primary reference person to address these topics. However, a third (32.8%) of FP were unfamiliar with information thereof. Some were unsatisfied regarding timely updates on their patient’s treatment course via discharge letters (25.0%) or phone calls (36.2%). FP would like to dedicate more consultation time addressing nutrition and PA than they currently do (*p* < 0.001).

**Conclusion:**

Communication btween healthcare practitioners about mutual cancer patient’s treatment must be improved, e.g. utilising electronic communication to quicken correspondence. Acquisition of information on nutrition and PA in cancer patient care needs to be facilitated for FP, approachable by compiling reliable information and their sources. Involvement of FP in structured treatment programs could benefit cancer patient care.

**Trial registration number:**

(May 7, 2021): 2021–2149-Bef.

**Supplementary Information:**

The online version contains supplementary material available at 10.1007/s00432-022-04233-5.

## Introduction

For most people, a cancer diagnosis is an incisive life event. Not only does cancer pose a mortal threat, but also its treatment can cause psychological, social, and physical effects impairing quality of life (QoL) and subjective wellbeing (Artherholt and Fann 2012; Brown et al. 2012; Ferlay et al. 2015; Leduc et al. 2017; Lewandowska et al. 2020). Various cancer- and treatment-related symptoms affect the patient’s nutritional and physical status (Bower et al. 2009; Garabige et al. 2007; Lee et al. 2016; Martin et al. 2008). In this regard, malnutrition is reported to occur in up to 80% of cancer patients (Lim et al. 2012; Montoya et al. 2010; Wie et al. 2010). In turn, malnutrition leads to adverse consequences such as a poor survival, reduced functional status, and reduced QoL (Capuano et al. 2010; Datema et al. 2011; Jager-Wittenaar et al. 2011; Mouri et al. 2018; Norman et al. 2010; Pressoir et al. 2010) Similarly, weight loss commonly occurs in cancer patients and increases mortality while reducing QoL (Antoun et al. 2010; Argiles et al. 2014; Awad et al. 2012; Kimura et al. 2015). Additionally, more than 50% of cancer patients report symptoms which impact their nutrition, such as appetite, early satiety, dysphagia, xerostomia, nausea, vomiting, and diarrhoea (Deftereos et al. 2021; Trajkovic-Vidakovic et al. 2012). Further restraints include a reduced physical functioning, exercise intolerance, dyspnoea, and fatigue (Maddocks 2020; Trajkovic-Vidakovic et al. 2012). It has been shown that most of these symptoms among many others are amendable to supportive nutritional and physical activity (PA) interventions, which includes prescription of nutrients and exercise, but also counselling on these topics (Arends et al. 2017; Avancini et al. 2020; Brown et al. 2012, 2009; Bye et al. 2020; Cormie et al. 2017; Edvardsen et al. 2015; Hilfiker et al. 2018; Lee et al. 2016; Maddocks 2020; McTiernan et al. 2019; Mouri et al. 2018; Sarwer et al. 2009; Scott & Tharmalingam 2019; Uster et al. 2018).

Since nutrition and PA are modifiable by the patient, they allow cancer patients to take part more actively in their cancer treatment. Many patients are motivated to do so, e.g. by searching for information or participating in exercise programs (Keinki et al. 2016; Maddocks et al. 2009; Maschke et al. 2017; van Veen et al. 2015). Various healthcare practitioners (HCP) participate in counselling and promoting nutrition and PA in cancer patients, such as oncologists, family physicians (FP) and other physicians, nurses, dietitians, and physical therapists (Dean et al. 2019; Halilova et al. 2019; Maschke et al. 2017; van den Berg et al. 2010; van Veen et al. 2020; van Veen et al. 2018). Yet, every second cancer patient is in need of information regarding beneficial lifestyle behaviour, and a high proportion is still malnutritioned or insufficient physically active (Deftereos et al. 2021; Erickson et al. 2018; Leach et al. 2015; Maschke et al. 2017; Philip et al. 2014; Pilotto et al. 2022; Zhou et al. 2022).

To improve counselling on nutrition and PA, multilevel strategies involving different HCP, including the FP, have been suggested (AuYoung et al. 2016; Hübner et al. 2013). FP substantially contribute to cancer patient care by coordinating care, managing comorbidities, promotion of health and self-management, and scheduling follow-up visits (Jefford et al. 2020; Lang et al. 2017). Furthermore, patients view FP as reliable and preferred source of health information, and they put major trust in them (Harris et al. 2004; Hudon et al. 2013; Wattanapisit et al. 2018). This predestines FP to act as reference person on the first level of the multilevel strategy, conveying basic information on nutrition and PA in cancer patients, and coordinating referrals for individual counselling on the second level. In fact, FP already give advice on these topics in other diseases like diabetes mellitus and hypertension, which is grounded on similar fundamental principles (Bantle et al. 2008; Booth and Nowson 2010; Brauer et al. 2012; Castro et al. 2015; Harris et al. 2004; Rock et al. 2012; Sigal et al. 2018; Wattanapisit et al. 2018). However, data investigating FPs attitude and involvement in nutritional and PA counselling in cancer patient care are scarce (Pilotto et al. 2022; Waterland et al. 2020). In this study, we aim to gain insight on FPs perception of nutrition and PA in cancer patient care and assess barriers and steps to improve their counselling on these topics.

## Methods

### Declarations

This study was approved by the Ethics Commission of the University Hospital of Jena (Reg.-Nr. 2021–2149-Bef). All participants gave informed written consent.

### Family physician interviews

#### Study design and interview guide

To explore what challenges FP face in conveying advice on nutrition and PA in cancer patients, we chose a qualitative phenomenological research approach. We conducted semi-structured interviews and categorized them using qualitative content analysis according to Mayring (Mayring 2015). The COREQ and SRQR guidelines were utilised to report on the findings (O'Brien et al. 2014; Tong et al. 2007).

The interview questions and structure were designed by the corresponding author, who had no prior experience in qualitative research, to explore the perception of FP of nutrition and PA in cancer patients. The questions were revised by the supervising author, who has expertise in the fields oncology, nutrition, PA, and qualitative research. After each interview, the FP were asked to evaluate the interview questions regarding their validity. The interview questions are appended in Supplement A: Interview Questions.

#### Recruitment of family physicians and data collection

Physicians who currently practised family medicine in Germany were eligible to participate. Only FP who attended to at least one cancer patient per week were included. A purposive sampling technique was used to acquire participants. A FP in Paderborn and Jena, respectively, helped to obtain participants. A total of nine FP were contacted, seven via telephone and two face-to-face. Six of them practised in Paderborn, North Rhine-Westphalia, and three in Jena, Thuringia (Germany). Seven FP were interested in the study, two of them later withdrew their assent. Reasons for declining participation were not recorded.

Over a period of 3 months from February to April 2021, five semi-structured interviews were conducted with FP by the corresponding author. During each interview, solely the interviewer and one FP were present. Four of the FP practised in Paderborn and one in Jena. One interview took place in the physician’s private practise, two at their respective homes, two more were carried out through phone. The interviews lasted on average 40 min. The participants received no monetary consideration. Supplement B: Flow Chart Family Physician Interviews summarises the development of the interview guideline and the recruitment of participants.

#### Data preparation and analysis

The interviews were recorded using a digital device. They were transcripted and anonymised with Microsoft Word (Microsoft 365 Apps for Enterprise). Using Microsoft Excel (Microsoft 365 Apps for Enterprise), the corresponding and supervising author performed qualitative content analysis according to Mayring (Mayring 2015), as seen in Supplement C: Flow Chart Interview Analysis.

### Family physician survey

#### Questionnaire design

A pilot questionnaire was developed by the corresponding author on basis of aforementioned semi-structured interviews to consolidate their results. The questionnaire was revised by the supervising author. The pilot version was tested once and adapted accordingly for length and comprehension among nine purposively sampled FP. Their demographics were similar to the sample population. Their answers are not included in the analysis. The final questionnaire consisted of 49 questions divided into 4 main sections:

- Demographic data including cancer patient consultations
- The topics *nutrition* and *PA* in cancer patient consultations
- Sources of information for FP on nutrition and PA in cancer care
- FP's perception of steps to improve a healthy lifestyle in cancer patients

We primarily used closed questions with lists of possible answers in form of Likert scales. Semi-open questions offered the participants space to add and elaborate answers.

#### Recruitment of family physicians and data collection

German-speaking and currently practising FP were eligible to participate. Using a listing of FP from an online service of the Association of Statutory Health Insurance Physicians (KV), a cross-sectional convenience sample of 150 FP was drawn. Ninety-three (62.0%) were successfully contacted by phone, and 49 (32.7%) agreed to consider participation. Eighteen of these completed the questionnaires (response rate 12.0%). Additionally, one FP helped to distribute the questionnaire by word of mouth and e-mail. In this way, further 46 FP completed questionnaires. The questionnaire was accessible online via Soscisurvey from October 2021 to March 2022. Over the same period, print versions were passed on to aforementioned general practices. Participation was voluntary and anonymous. Reasons for declining participation were not recorded.

#### Data preparation and analysis

Answer forms stating no participation in family medical care were excluded. Answer forms missing at least 30% answers were excluded (‘non-responders’). Data were collected and analysed using IBM SPSS Statistics 27. During analysis, missing values were excluded listwise. Mann–Whitney *U* tests were performed to analyse differences in central tendencies between two groups and significances tested asymptotically (*Z:* standardised score, effect size *r* =|*Z|* /√*n*). Effect sizes were estimated to be small (0.1), medium (0.3) or large (0.5) according to Cohen (Cohen 1988). *p* values smaller than 0.05 were considered significant.

## Results

Representative interview statements have been selected to portray the results. Additional quotes can be found in Supplement D: Interview Results.

### Samples

A total of five FP were interviewed; two of them were female, three were male. All of them practised in a city (more than 100.000 habitants). Four had at least 10 years’ experience of general practice. The sample is described within Supplement B: Flow Chart Family Physician Interviews.

A total of 61 questionnaires were included in the questionnaire analysis. Male FP participated slightly more often than female (55.7% or 44.3%), their ages ranging from 33 to 74 years (mean value 53.71 ± 9.26 years). Most of them practised as FP for more than 20 years (46.0%), 31.1% between 10 and 19 years, and 23.0% for less than 10 years. The demographic data are shown in Table 1.

**Table 1:** Characteristics of the trial population (*N* = 61)

Criteria	Number	Percentage (%)
Gender		
Female	27	44.3
Male	34	55.7
Age (years)		
Under 40	7	11.5
40—49	8	13.1
50—59	31	50.8
60—69	11	18.0
Over 70	2	3.3
n.a	2	3.3
Practising as family physician for … years		
Less than 5	9	14.8
5–9	5	8.2
10–19	19	31.1
20–25	14	23.0
More than 25	14	23.0
Type of doctor’s practice		
Single practice	22	36.1
Group practice	36	59.0
Medical care centre	3	4.9
Number of inhabitants in the community of the physician’s practice		
1.000 – 9.999	3	4.9
10.000 – 99.999	26	41.6
100.000 – 500.000	30	49.2
More than 500.000	1	1.6
n.a	1	1.6
Attending to number of cancer patients (per month)		
< 10	19	31.1
11—20	19	31.1
> 20	21	34.5
n.a	2	3.3

### Importance of nutrition and physical activity in cancer patients

All five FP agreed on the high relevance of nutrition and PA in cancer patients. Likewise in the survey, close to all FP valued nutrition and physical as (very) important both during (*n* = 53, 91.4%) and after (*n* = 59, 100%) cancer therapy. Eighty-five percent (*n* = 51, 85.0%) of the FP reported to talk about these topics with their cancer patients.

For this fundamental theme, three sub-themes were identified: (a) constraints in nutrition and PA, (b) nutrition and PA as supportive treatment and (c) habits.

“Cancer patients lose weight unexpectedly. [During treatment,] they become weak. Some cannot walk a staircase. Some cannot taste or swallow their food.” (No. 3, Pos. 18ff)

“There is no cure without operation, radio- or chemotherapy. Nutritional or exercise therapy does not heal the cancer. But without it, recovery might be impaired.” (No. 4, 99ff)

“Someone who likes to eat sweet will not suddenly omit sweets or meat. In addition, dining can be a ritual and unifying experience. In a family of connoisseurs, it is a challenging task to adhere to a diet.” (No. 5, Pos. 245ff)

“Patients whose exercise routine is limited by their disease regain quality of life when I tell them to exercise like they are used to. Not quite as vigorous, but to the best of their ability.” (No. 3, 256ff)

### Responsibilities of participants in cancer care

While many people and institutions took part in cancer care, the interviewed FP highlighted the responsibilities for following participants in unison: (a) oncologists, (b) rehabilitation institutions, (c) FP, (d) relatives, and (e) politics.

“The oncologist usually conducts the […] therapy. I think he is pivotal in the acute treatment of the patient.” (No. 1, Pos. 61f)

“During acute therapy, the patient fights to survive and is hardly accessible [to information on nutrition and physical activity]. During rehabilitation, they learn that exact information.” (No. 1, Pos. 116ff)

“It is our [the family physicians’] duty to give lifestyle advice. The family physician attends to the whole patient and sees him on a regular basis.” (No. 1, Pos. 69f)

“The relatives take care of the patient and support him/her. Sometimes I call them to request their support for the patient.” (No. 4, Pos. 57)

“Politics clearly influences patient care. But political decisions are ultimately about money. (…) The political intent does hardly care [for patient care].” (No. 5, Pos. 82ff)

**Fig. 1:**
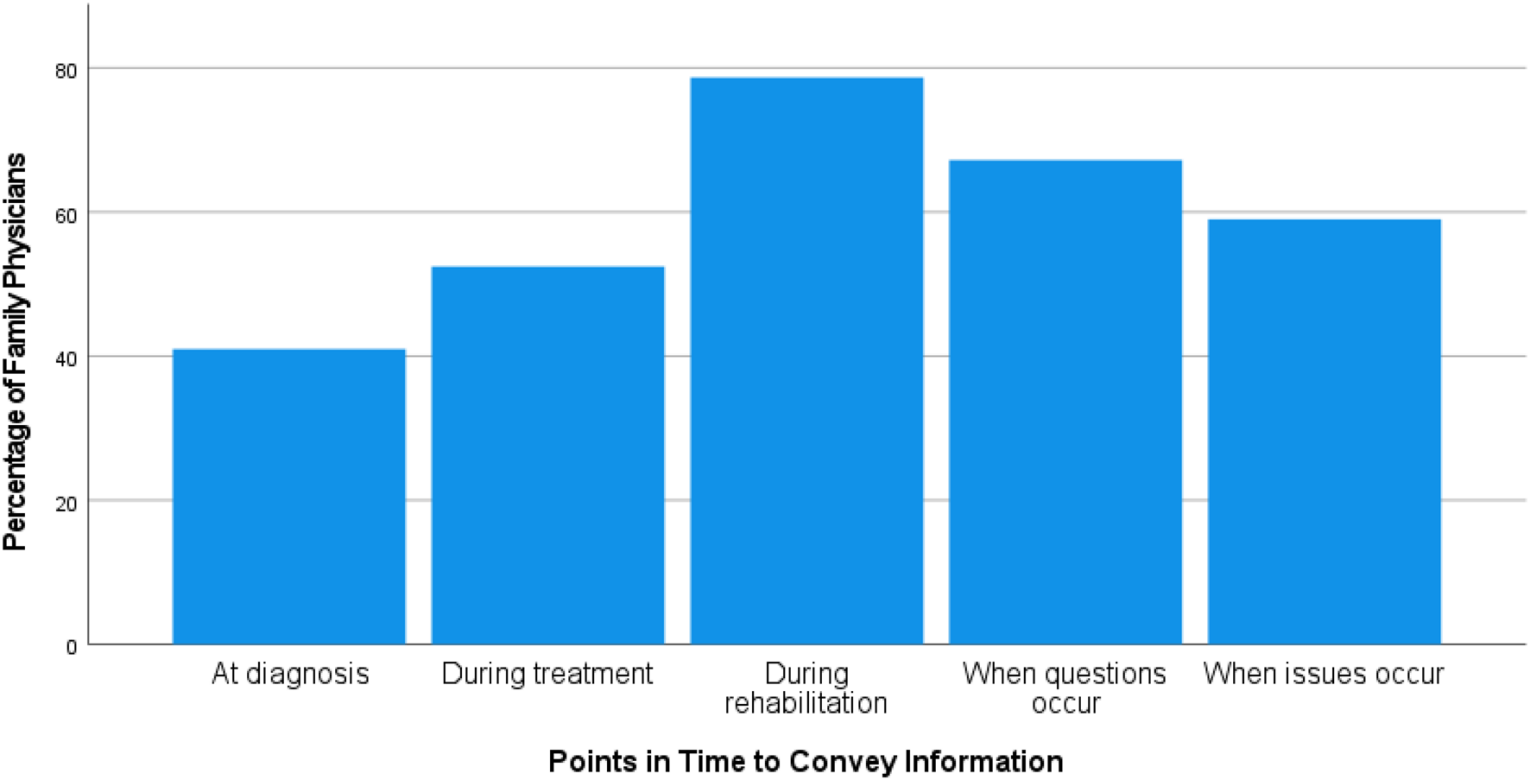
Preferred points in time to convey information on nutrition and physical activity (*N* = 61, several answers possible)

In the interviews, the FP suggested that rehabilitation would be the best point in time to convey information on nutrition and PA to cancer patients. The questionnaire yielded concordant results, as can be seen in Fig. 1.

**Fig. 2:**
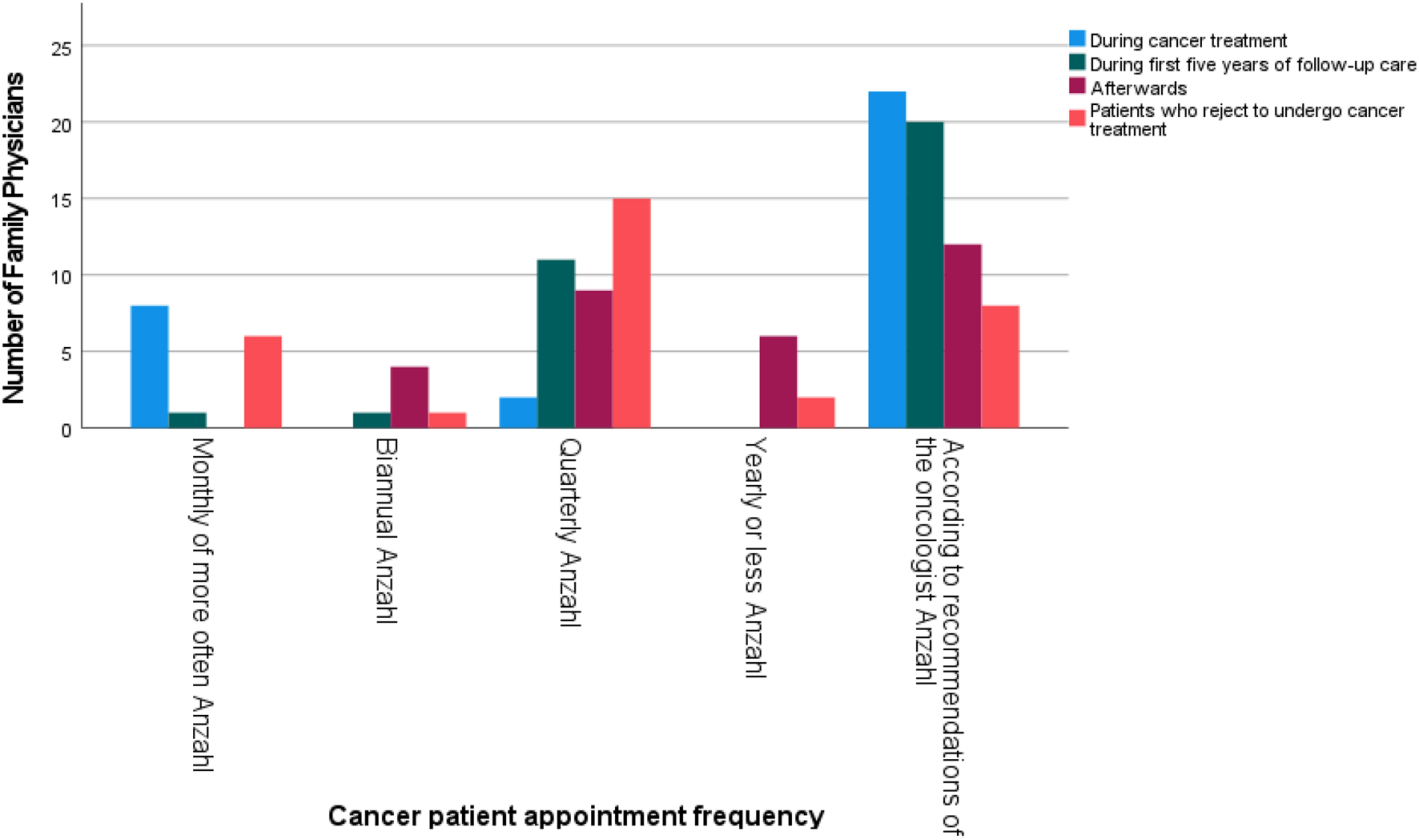
Frequencies of family physicians appointing cancer patients in different treatment phases (*N*_afterwards_ = 31, *N*_treatment, rejecting_ = 32, *N*_follow-up_ = 33)

Two thirds (*n* = 40, 65.6%) of the FP viewed themselves as primary reference persons to address nutrition and PA in cancer patients, and they reported to see cancer patients on a regular basis. However, they hardly had access to the patient during his initial treatment. In the survey, the FP reported to attend to more cancer patients in follow-up care than during their treatment (*Z* = − 3.264, *r* = 0.301, *p* = 0.001). More than half of the physicians (*n* = 33, 56.9%) arranged appointments with cancer patients on a regular basis. The frequencies cancer patients are summoned in different phases of their treatment are shown in Fig. 2. FP arranged appointments with cancer patients during their treatment more frequently than in the first 5 years of follow-up care (*Z* = − 3.413, *r* = 0.712, *p* = 0.001), usually monthly or more often. Accordingly, patients were summoned more frequently in their first 5 years of follow-up care than the years after (*Z* = − 2.798, *r* = 0.495, *p* = 0.005), usually quarterly.

### Challenges for family physicians in cancer patient care

The interviewed physicians reported on issues they faced providing cancer patient care. Three sub-themes stood out: (a) unsatisfactory communication with other healthcare providers, (b) unfamiliarity with information sources, and (c) lack of time to inform themselves or their patients.

*“[As family physician,] you get left out (…). Neither do I receive updates from the specialist I referred to (…), nor from the clinicians and rehabilitation institutions. Sometime later, the patient arrives with his treatment done (…), and I don’t know anything of what happened. (…) Although to be honest, in some cases the communication does work well.” (No. 3, Pos. 110ff).*

“I don’t know of any such information [on nutrition and physical activity in cancer patients].” (No. 1, Pos. 184)

“From time to time, I try to browse the internet [for information]. But quickly I get into a lost position, not knowing which information to rely on.” (No. 5, Pos. 154ff)

“We don’t have time to counsel on nutrition and physical activity.” (No. 2, Pos. 175)

“We spend too many resources [e.g. time, personnel, material] on bureaucratic efforts, without use for anybody, and particularly without use for patients.” (No. 1, Pos. 307)

In the interviews, timely communication between healthcare providers has been criticised repeatedly. Evaluating the communication between clinicians and FP in the survey, in general the latter were satisfied with discharge letters and phone calls. However, twenty-five (*n* = 15, 25.0%) and thirty-six percent (*n* = 21, 36.2%), respectively, reported that they did not receive letters or phone calls in a timely manner.

**Fig. 3:**
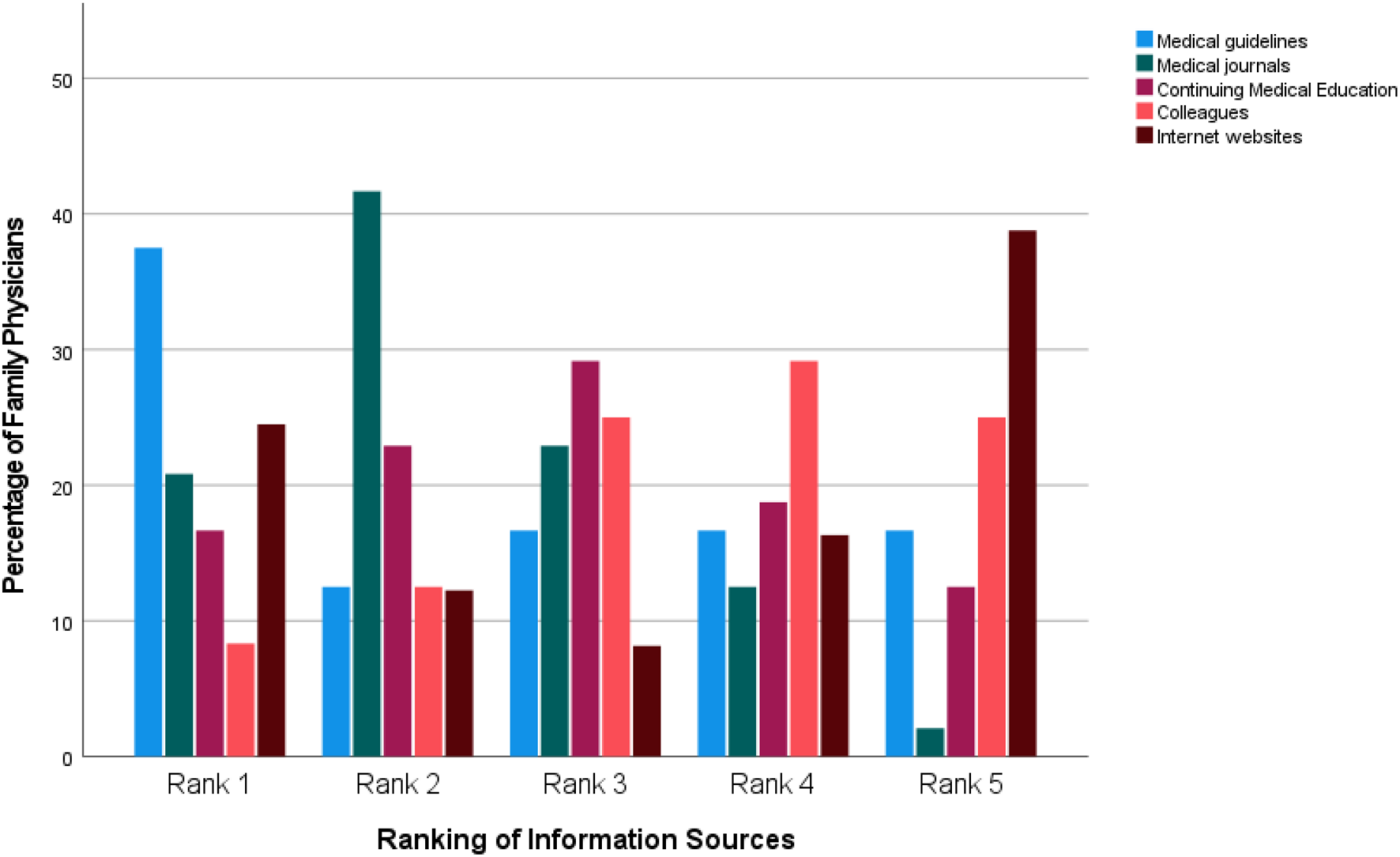
Sources for information on nutrition and physical activity in cancer patients of family physicians (Rank 1: utilised the most; Rank 5: utilised the least; *N* = 48)

The interviewed FP were not familiar with specific information on nutrition and PA in cancer patients, or where to find such information. In the survey, one-third of the FP (*n* = 19, 32.8%) were unfamiliar with sources on these topics. While the internet has been considered as information source, they had differing opinions on its reliability. In the survey, the FP reported that if they searched for such information, they would utilise medical journals (rank 1 and 2: *n* = 30, 62.5%) and guidelines (rank 1 and 2: *n* = 24, 50%) the most. Browsing the internet was ranked both high by 18 (rank 1 and 2: 37.5%) and low by 26 physicians (rank 4 and 5: 54.2%). The rankings of different information sources are reported in Fig. 3.

The FP raised concern in the interviews that they lacked time to inform their cancer patients on nutrition and PA. In the survey, the FP reported that they would like to dedicate more time during cancer patient consultation addressing nutrition and PA than they currently did (*Z* = − 6.237, *r* = 0.565, *p* < 0.001).

For example, the interview respondents deemed bureaucratic efforts in general too time-consuming. The majority of questionnaire participants (*n* = 44, 72.1%) experienced too much bureaucratic efforts to initiate and sustain programs to encourage a healthy lifestyle, such as Disease Management Programs.

### Approaches to improve lifestyle counselling by family physicians

All five FP proposed ideas on how to improve the involvement of family physicians in counselling on nutrition and PA to cancer patients. The following three approaches were each considered by at least two FP.

“I would sometimes like an update by phone; or a discharge letter which doesn’t arrive four to five months late or which is directly handed to the patient.” (No. 2, Pos. 46ff)

“Validated papers or review articles that show which nutritional and exercise is significantly improving the patient’s wellbeing would be useful. But I guess those do exist – if you know where to search for them.” (No. 5, Pos. 303ff)

“It would be really easy to improve nutrition and physical activity in cancer patients. In Germany, we already have Disease Management Programs. Part of these structured treatment programs are not only regular follow-up appointments, but also patient education and counselling.” (No. 1, Pos. 269ff)

“Disease Management Programs are economic stimulus programs for physicians with little benefit for the patient. Follow-up care would work fine without them.” (No. 5, Pos. 257ff)

In the interviews, differing opinions on implementing structured treatment programs such as Disease Management Programs (DMP) to improve lifestyle counselling by FP have been disclosed. The survey shows similar results with either half of FP advocating (*n* = 29, 48.3%) or rejecting (*n* = 31, 51.7%) the proposal of establishing DMP for additional types of cancer.

## Discussion

To our knowledge, this is the first study capturing the perception of FP regarding nutrition and PA in cancer patient care in Germany. The results indicate that they acknowledge the importance of these lifestyle factors in cancer treatment and frequently address them. However, they have little knowledge of reliable information sources hereof. Since FP feel responsible to give lifestyle advice, counselling could be improved by providing compiled information and continuous education on nutrition and PA. Furthermore, communication between FP and other healthcare providers needs improvement to keep FP up to date and integrate them into structured treatment programs.

Family physicians convey the importance of nutrition and physical activity.

The importance and benefit of nutrition and PA in cancer patients are well established. Congruent to other data, almost every FP in our study acknowledges the importance of nutrition and PA during cancer treatment and follow-up care as these topics occur in various ways (Pilotto et al. 2022; Waterland et al. 2020): weight loss as first cancer symptom; cancer- and treatment-related restraints in everyday tasks, but also worsening their outcome; alleviation of symptoms, outcome, and QoL by means of nutrition and PA (Arends et al. 2017; Edvardsen et al. 2015; Lee et al. 2016; Mouri et al. 2018). To adapt a beneficial lifestyle requires the patient to pick up old or new habits, which can constitute either a facilitator or barrier (Pilotto et al. 2022). Beyond the treatment phase, adhering to a healthy lifestyle has been shown to improve the patient’s QoL and reduce cancer relapses (Arends et al. 2017; Cormie et al. 2017; Mouri et al. 2018). The majority of FP feel responsible to convey this importance of nutrition and PA to cancer patients. Eighty-five percent report addressing these topics with their cancer patients. This perception is congruent to previous studies on opinions of cancer care providers (Jefford et al. 2020; Pilotto et al. 2022; Sisler et al. 2016; Waterland et al. 2020).

### Family physicians lack knowledge and time for counselling.

Many HCP are involved in nutritional care and exercise therapy, such as oncologists, FP, dietitians, physical therapists, and nurses. While acknowledging their responsibilities in lifestyle counselling, many report lacking knowledge and time to provide advice on nutrition and PA to cancer patients, as is also true in our study (Avancini et al. 2020; "EFAD and ESDN Oncology Statement Paper on the Role of the Dietitian in Oncology," 2021; Kirbiyik and Ozkan 2018; Massoud et al. 2021; Pilotto et al. 2022; van Veen et al. 2017; Waterland et al. 2020).

As shown in previous studies, FP reported a lack of FP-specific resources, programs, and journal articles to educate themselves on nutrition and PA in cancer patient (Ciarlo et al. 2016; Waterland et al. 2020). Concordantly, one-third of our sample were not familiar with any sources on these topics, indicating a need for improvement. As nutrition and PA in cancer patients are among the information needs of FP, such information should be readily accessible, brief, concise, and reliable (Ciarlo et al. 2016). Although the internet could provide an opportunity to fulfil these requirements, in general, the FP do not use it more than other sources of expert information. This mirrors the ambivalence towards the internet as source of information: whereas almost 40% of FP would utilise it the most, more than 50% would use it the least; on one hand, the internet is readily accessible, but it is time-consuming identifying reliable information with a high percentage of websites providing false information or information of low quality (Ogasawara et al. 2018). To exploit its full potential, a compilation of reliable and trustworthy online resources for FP should be established, as they do exist, e.g. in form of articles, guidelines, and brochures (Brown et al. 2012; Deutsche Krebsgesellschaft 2022; Leitlinienprogramm Onkologie: Deutsche Krebsgesellschaft et al. 2021; Tajan & Vousden 2020).

### Integration of family physicians in structured treatment programs

Even though FP attend to cancer patients on a regular basis, the general practice setting lacks the time required to affect lifestyle behaviours in cancer patients (McPhail and Schippers 2012; Waterland et al. 2020). It is rather the shared responsibility of many HCP to encourage a beneficial lifestyle in cancer patients. As also concluded by previous authors, HCP should receive continual education on nutrition and PA in cancer patients to reinforce awareness of its importance (Erickson et al. 2018). Furthermore, specialists in nutrition and PA are available, but structural referral pathways appear to be missing (Erickson et al. 2018; Pilotto et al. 2022). Since FP contribute substantially to coordinating cancer patient care, such pathways should be established and FP integrated (Druel et al. 2020; Edbrooke et al. 2020; Hickner et al. 2007; Jefford et al. 2020; Sisler et al. 2016). This could be realised through structured treatment programs like Disease Management Programs (DMP), as suggested by FP. DMP are structured treatment programs for chronically ill patients. They rest on evidence-based medicine and exist in Germany for various diseases like breast cancer, diabetes mellitus, and coronary heart disease (Kassenärztliche Bundesvereinigung). Patients enrolled in DMP regularly visit HCP like their FP, and they receive information on their disease. Information on nutrition and PA should become part of DMP for cancer. Since attitudes of FP towards DMP are varying, their concerns regarding bureaucratic efforts and challenges in documentation requirements must be considered, and the benefit of DMP on patient care quality must be subject to continuous scrutiny to ensure applicable and effective health programs (Wangler and Jansky 2021).

### Family physicians are missing timely updates

FP conduct a major part of cancer patient care. As our study indicates in line with other data, they are more intensely involved at the time of diagnosis and in follow-up care (Hickner et al. 2007; Waterland et al. 2020). Currently, they are missing out on timely updates on their patients’ status, and they would like to be more integrated during acute cancer treatment (Easley et al. 2017; Hickner et al. 2007; Waterland et al. 2020). To ultimately achieve this, interdisciplinary communication is mandatory. While the FP were generally content regarding interdisciplinary communication, they criticised its timeliness, leading to missing information on their patient’s current treatment. This perception is in line with studies on communication between primary and secondary HCP (Easley et al. 2016, 2017; McConnell et al. 1999; Suija et al. 2016; Vermeir et al. 2015). Poor communication can lead to various negative outcomes, such as wasting the physician’s time and lengthening the patient’s stay, subsequently causing medical errors, stress, and frustration (Vermeir et al. 2015; Waterland et al. 2020). Utilising electronic communication to exchange information between primary and secondary care providers and mutual feedback yield the potential to approach this issue (Pannell and Tyrrell-Price 2017; Vermeir et al. 2015).

### Limitations and future research

Due to the small sample size of the interviews, the results are scarcely applicable to FP at large. All interviewed physicians practised in cities with more than 100.000 inhabitants. Since only 32% of the German population live in such cities, the transferability of this study’s results to physicians practising in more rural areas is limited (Europäische Kommission eurostat 2021). Besides, saturation of aspects mentioned by the participants has not necessarily been reached, therefore, further notable remarks may have been missed. Moreover, the non-random acquisition of interviewees is susceptible to causing bias. Finally, socially desirable answers cannot be excluded.

The FP recognised the significance of lifestyle in cancer prevention, but that aspect is beyond the scope of this study.

The questionnaire was not validated but developed by experts including oncologists, FP, and statisticians based on preceding qualitative research, literature research, and experience in their field of work. Due to the small sample size, the results are not necessarily representative or applicable to FP at large. Furthermore, a selection bias may have caused physicians to preferably participate in the study if they are already familiar with nutrition and PA in cancer patients. Socially desirable answers cannot be excluded. Future research on these topics should aim to recruit a larger randomised sample size to reduce bias-susceptibility and improve external validity, enhancing transferability to FP at large.

## Conclusion

Since FP lack time to look to educate themselves, information should be readily available, concise, and reliable. The internet could fulfil these criteria; however, as it is vast and ever-growing, navigating can be confusing. Compilating existing information sources on these topics such as medical guidelines, journal articles, and other websites, could help them to acquire knowledge and counsel cancer patients confidently in this regard. FP desire improvements in interdisciplinary communication and want to be integrated more during cancer treatment. Development of structured treatment programs could address the latter and provide benefits to patient care and outcomes. This would facilitate to involve FP into giving lifestyle advice to cancer patients as they do feel responsible to do so.

## Supplementary Information

Below is the link to the electronic supplementary material.

Supplementary file1 (DOCX 2550 KB)

## Data Availability

Data available on request due to privacy restrictions. The data that support the findings of this study are available on request from the corresponding author, S. J. Boesenecker. The data are not publicly available due to their containing information that could compromise the privacy of research participants.
