# Supplementary material for: How can counselling by family physicians on nutrition and physical activity be improved: trends from a survey in Germany
Source: J Cancer Res Clin Oncol. 2022 Aug 6;149(7):3335–47. doi: 10.1007/s00432-022-04233-5 (PMC10314832; doi:10.1007/s00432-022-04233-5)

## Supplement A: Interview Questions

| Category | Questions |
| --- | --- |
| General Questions | - How often do you see cancer patients? |
|  | - How secure do you feel about the topics *nutrition and physical activity in cancer care*? |
|  | - Who do you perceive as actors in cancer care? What are their responsibilities? |
|  | - What is the best point in time to convey information on nutrition and physical activity in cancer treatment to cancer patients? |
|  | - What are currently strengths / weaknesses in cancer care? |
| Questions on Nutrition / Physical Activity (PA)  (Whole question block for nutrition first, then repeat questions for PA) | - How do you estimate the relevance of nutrition / PA in cancer treatment? |
|  | - Where do your patients / do you receive information on nutrition / PA in cancer treatment? |
|  | - Whose responsibility is it to counsel cancer patients regarding nutrition / PA? |
|  | - What are enablers / barriers for cancer patients to realise a healthy diet / regular PA? |
| Closing Questions | - What if: You want to improve a healthy lifestyle in cancer patients. If you could change or establish something what- and however you like, what would you do? |
|  | - Meta-level: Please evaluate the questions: What question would you have liked to have been asked that was missing? Do you think the questions were reasonable? Have there been superfluous questions? Please elaborate critique. |
|  | - Any other thoughts you want to share? |

## Supplement B: Flow Chart Family Physician Interviews

Qualitative, phenomenological: Semi-structured interviews, open-ended questions

Purposive sampling

Corresponding author: no prior experience in qualitative research

Places:

- 1 at participant’s private practice
- 2 at participant’s home
- 2 by phone

9 family physicians eligible:

- 7 contacted via phone
- 2 contacted face-to-face
- 2 not interested
- 2 withdrew assent

Inclusion criteria:

- family physician
- currently practising in Germany
- attending to at least one cancer patient per week

Conducted by the corresponding author from February to April 2021

**5 family physicians participating,** giving written informed consent

Supervising author: expertise in oncology, nutrition, physical activity, qualitative research

**Recruitment**

**Questionnaire Design**

**Research Approach**

- Interviewer and family physician present
- on average 40 minutes per interview
- digital recording
- no monetary consideration

| Gender | Number |
| --- | --- |
| Female | 2 |
| Male | 3 |
| Age (years): |  |
| 41 - 50 | 1 |
| 51 - 60 | 4 |
| Experience (years): | |
| 5 – 10 | 1 |
| > 10 | 4 |
| All five practised in a city (> 100.000 inhabitants) | |

## Supplement C: Flow Chart Interview Analysis

Select representative statements to reflect the perceptions of family physicians

Assign statements to the derived categories

**Step 8: Interpretation**

**Step 7: Compilation**

Summarize categories (second reduction)

Review and revise categories

Abstract essence of each paraphrase and derive categories (first reduction)

**Step 6: Reduction**

**Step 5: Paraphrasing the text material**

Listen to audio material iteratively, read text material simultaneously

Perceive statements and viewpoint in their entirety

**Step 4: Familiarisation with the data**

**Step 3: Analytical approach**

Inductive category formation

How can counselling by family physicians on nutrition and physical activity in cancer patients be improved?

**Step 2: Research question**

Bracketing authors’ perceptions, thoughts, and preconceived notions

Identify origin and context, transcribe digital recordings (*Microsoft Word*)

**Step 1: Identifying data**

## Supplement D: Interview Results

| Themes & Subthemes | Examples |
| --- | --- |
| Importance of nutrition and physical activity in cancer patients   - Constraints in nutrition and physical activity - Nutrition and physical activity as supportive treatment - Nutrition and physical activity habits | - “Cancer is a consuming disease. Patients suffer deficits in nutrition and musculature.” (No. 4, Pos. 96f) |
|  | - “Weight loss is often the first symptom, leading the patient to the family physicians. (…) Even an adequate cancer treatment will leave patients weaker than at the time of the initial diagnosis.” (No. 1, Pos. 146ff) |
|  | - “Cancer patients lose weight unexpectedly. [During treatment,] they become weak. Some cannot walk a staircase. Some cannot taste or swallow their food.” (No. 3, Pos. 18ff) |
|  | - “There is no cure without operation, radio- or chemotherapy. Nutritional or exercise therapy does not heal the cancer. But without it, recovery might be impaired.” (No. 4, 99ff) |
|  | - “In cases like intestinal cancer, nutrition is essential for survival. In other cases, nutrition is part of maintenance therapy rather than cancer therapy.” (No. 2, 56ff) |
|  | - “It is essential that cancer patients do not lose more weight. It does not need to be the healthiest diet. They just need calories so that they don’t slide into malnutrition.” (No. 3, Pos. 140ff) |
|  | - “Because of the cancer treatment, the patients lose even more weight. It is all the more important to subsequently conduct a profound nutritional and exercise therapy. (…) Otherwise, they can entail other conditions like risk of falling, leading to injuries which can be lethal.” (No. 1, Pos. 151ff) |
|  | - “Someone who likes to eat sweet will not suddenly omit sweets or meat. In addition, dining can be a ritual and unifying experience. In a family of connoisseurs, it is a challenging task to adhere to a diet.” (No. 5, Pos. 245ff) |
|  | - “For some it is convenient to watch TV rather than walk outside.” (No. 1, Pos. 260) |
|  | - “Patients whose exercise routine is limited by their disease regain quality of life when I tell them to exercise like they are used to. Not quite as vigorous, but to the best of their ability.” (No. 3, 256ff) |
|  | - “When patients believe that their cancer is gone, their adherence [to a healthy lifestyle] reduces.” (No. 1, Pos. 262) |
| Responsibilities of participants in cancer care   - Oncologists initiate and conduct acute therapy - Rehabilitation as preferred point in time to counsel lifestyle advice - Family physicians conduct follow-up care - Relatives provide support - Politics prioritise money over patient care | - “The oncologist usually conducts the chemotherapy. I think he is pivotal in the acute treatment of the patient.” (No. 1, Pos. 61f) |
|  | - “It is primarily oncologists who utilise new types of medication, e.g. immunotherapies. In this regard, they are kept up to date by their inner circles and conferences.” (No. 3, Pos. 11f) |
|  | - “In regard to decision making in treatment and progress monitoring, I give priority to the opinion of the oncologist over mine.” (No. 5, Pos. 38f) |
|  | - “Oncologists have plenty other work to do. (…) There is no time left to talk about what to put on the table.” (No. 3, Pos. 59ff) |
|  | - “I doubt that oncologists mention physical activity as part of cancer treatment.” (No. 2, Pos. 89) |
|  | - “Rehabilitation institutions are very important. (…) They can tailor lifestyle advice to the patient’s specific disease, which is of extreme importance.” (No. 1, Pos. 77ff) |
|  | - “I am grateful for the lifestyle advice (…) and psychological care provided to patients by rehabilitation institutions.” (No. 5, Pos. 31 & Pos. 73) |
|  | - “Counselling on nutrition and physical should take place during rehabilitation.” (No. 2, Pos. 35) |
|  | - “During acute therapy, the patient fights to survive and is hardly accessible [to information on nutrition and physical activity]. During rehabilitation, they learn that exact information.” (No. 1, Pos. 116ff) |
|  | - “I suspect a tumour and refer the patient [to a specialist].” (No. 3, Pos. 125) |
|  | - “The family physician obviously participates in follow-up care.” (No. 4, Pos. 40) |
|  | - “We follow up with symptomatic treatment.” (No. 2, Pos. 25) |
|  | - “It is our duty to give lifestyle advice. The family physician attends to the whole patient and sees him on a regular basis.” (No. 1, Pos. 69f) |
|  | - “I would feel responsible [to counsel on nutrition and physical activity] if I had well-founded knowledge.” (No. 5, Pos. 175) |
|  | - “Relatives in a stable family connection are worth a mint.” (No. 5, Pos. 100) |
|  | - “Clearly, the family provides psychological support. They organise the home environment and deal with the bureaucracy.” (No. 2, Pos. 28ff) |
|  | - “The relatives take care of the patient and support him/her. Sometimes I call them to request their support for the patient.” (No. 4, Pos. 57) |
|  | - “Relatives experience the patient at home when he/she feels really bad. They want to help, but sometimes they can just watch.” (No. 3, Pos. 83ff) |
|  | - “Sometimes, family members struggle to cope with the patient’s disease. Then I attend to them as well.” (No. 5, Pos. 94ff) |
|  | - “Politics clearly influences patient care. But political decisions are ultimately about money. (…) The political intent does hardly care [for patient care].” (No. 5, Pos. 82ff) |
|  | - “I doubt that politics care about follow-up care. They just want the expenses to be minimal.” (No. 4, Pos. 60ff) |
|  | - “Politics indirectly influences cancer patient care through legislation and remuneration.” (No. 3, Pos. 75) |
| Challenges for family physicians in cancer patient care   - Communication with other healthcare providers is unsatisfactory - Information sources are unfamiliar - Family physicians lack time to inform themselves or cancer patients | - “You get left out (…). Neither do I receive updates from the specialist I referred to (…), nor from the clinicians and rehabilitation institutions. Sometime later, the patient arrives with his treatment done (…), and I don’t know anything of what happened. (…) Although to be honest, in some cases the communication does work well.” (No. 3, Pos. 110ff). |
|  | - “The communication between the hospital and the attending family physician is abysmal.” (No. 2, Pos. 44) |
|  | - “The oncologists often discuss something with the patient, and I don’t get to know about it. I don’t know what treatment goals they want to accomplish or how they want to achieve them. When the patient comes to see me and asks for my opinion, I did not yet know of the treatment procedure in question.” (No. 4, Pos. 82ff) |
|  | - “I don’t know of any such information.” (No. 1, Pos. 184) |
|  | - “I will probably find something [on these topics] on the internet.” (No. 4, Pos. 129) |
|  | - “I did not yet search for information [on nutrition and physical activity in cancer patients] specifically. Because the multitude of new therapeutic options and their side effects are difficult for me to comprehend.” (No. 3, Pos. 194ff) |
|  | - “From time to time, I try to browse the internet [for information]. But quickly I get into a lost position, not knowing which information to rely on.” (No. 5, Pos. 154ff) |
|  | - “I don’t know of any such information.” (No. 1, Pos. 184) |
|  | - “It would be nice to know, where to look information up. But even then, it requires time [to read it].” (No. 3, Pos. 363) |
|  | - “You could buy a book. But you don’t have time [to read it].” (No. 1, Pos. 182f) |
|  | - “Family physicians need more time to spend on follow-up care.” (No. 4, Pos. 224f) |
|  | - “We don’t have time to counsel on nutrition and physical activity.” (No. 2, Pos. 175) |
|  | - “We spend too many resources [e.g. time, personnel, material] on bureaucratic efforts, without use for anybody, and particularly without use for patients.” (No. 1, Pos. 307) |
| Approaches to improve lifestyle counselling by family physicians   - Phone calls and quicker discharge letters - Review articles on nutrition and physical activity in cancer patients - Structured treatment programs | - “I would sometimes like an update by phone; or a discharge letter which doesn’t arrive four to five months late or which is directly handed to the patient.” (No. 2, Pos. 46ff) |
|  | - “The attending family physician should be more transparently informed about the patient’s treatment procedure, and quicker discharge letters are needed.” (No. 4, Pos. 92f) |
|  | - “Validated papers or review articles that show which nutritional and exercise is significantly improving the patient’s wellbeing would be useful. But I guess those do exist – if you know where to search for them.” (No. 5, Pos. 303ff) |
|  | - “It is important to have scientifically well-founded documents to show what [nutritional or exercise] interventions are reasonable. That could be professional brochures for patients. (…) I think, you’ve got to address this issue [conveying lifestyle advice to cancer patients] on a higher level than at family physician’s.” (No. 3, Pos. 354ff) |
|  | - “It would be really easy to improve nutrition and physical activity in cancer patients. In Germany, we already have Disease Management Programs. Part of these structured treatment programs are not only regular follow-up appointments, but also patient education and counselling.” (No. 1, Pos. 269ff) |
|  | - “Disease Management Programs are economic stimulus programs for physicians with little benefit for the patient. Follow-up care would work fine without them.” (No. 5, Pos. 257ff) |

## Supplement E: Study Questionnaire (German)


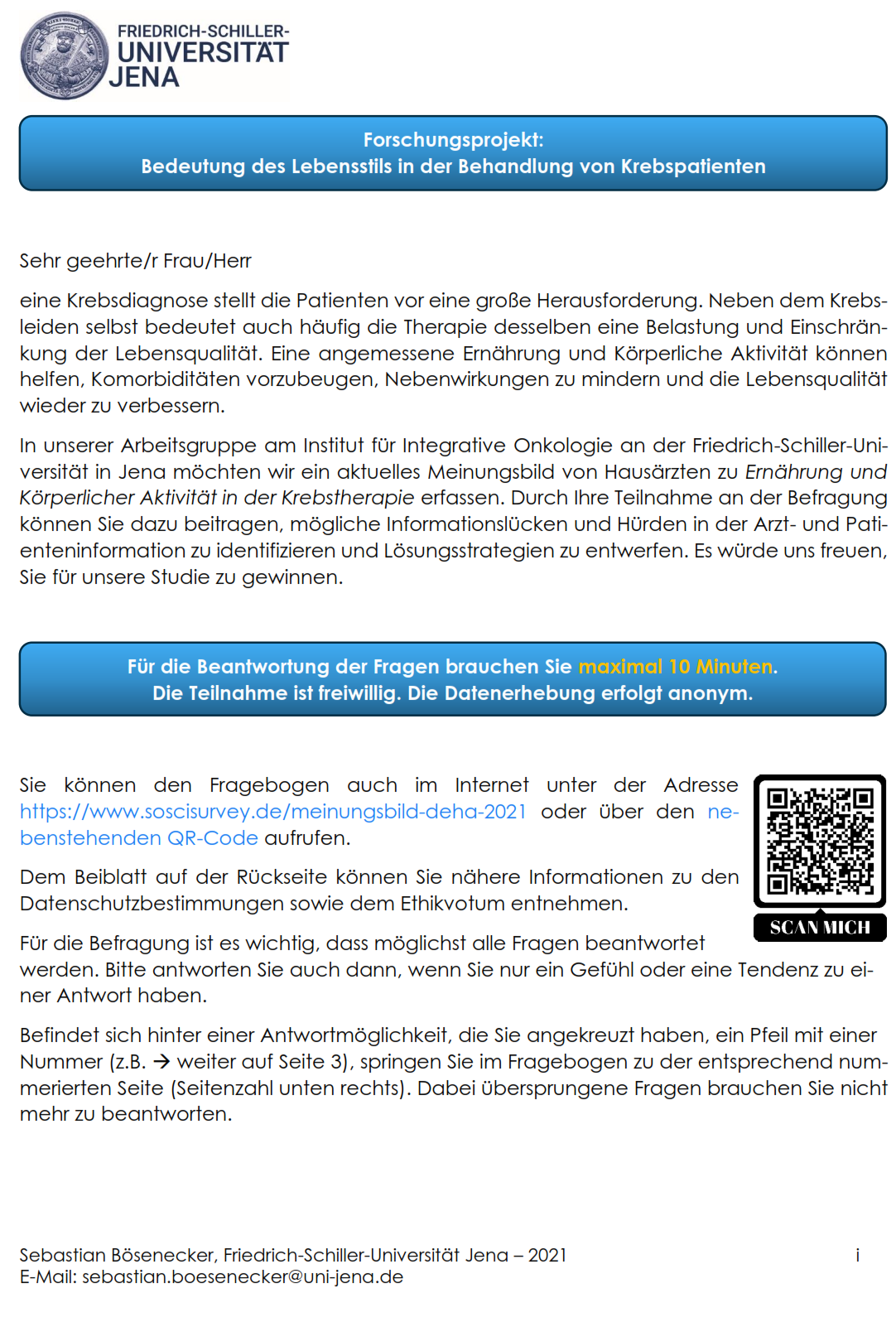

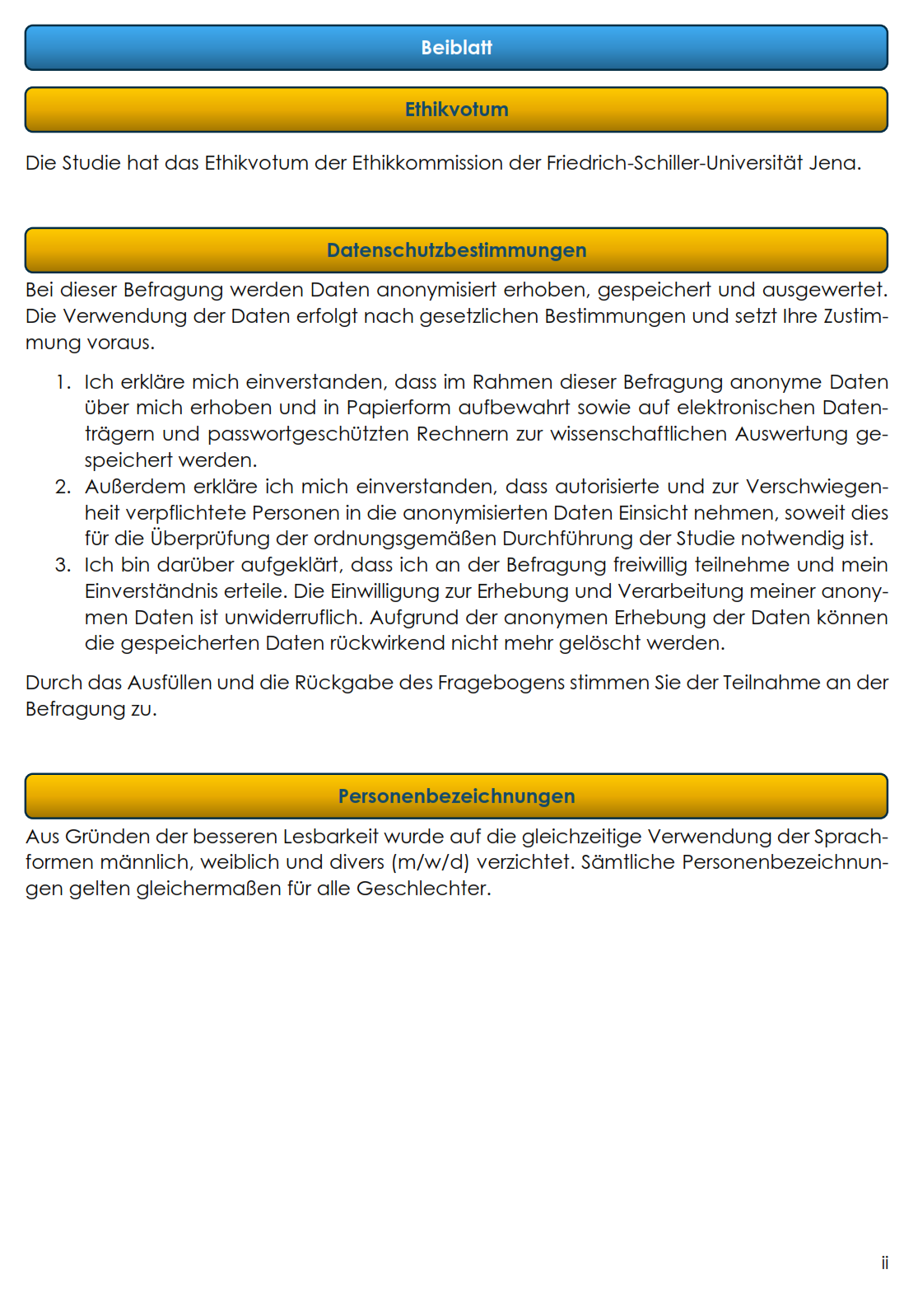

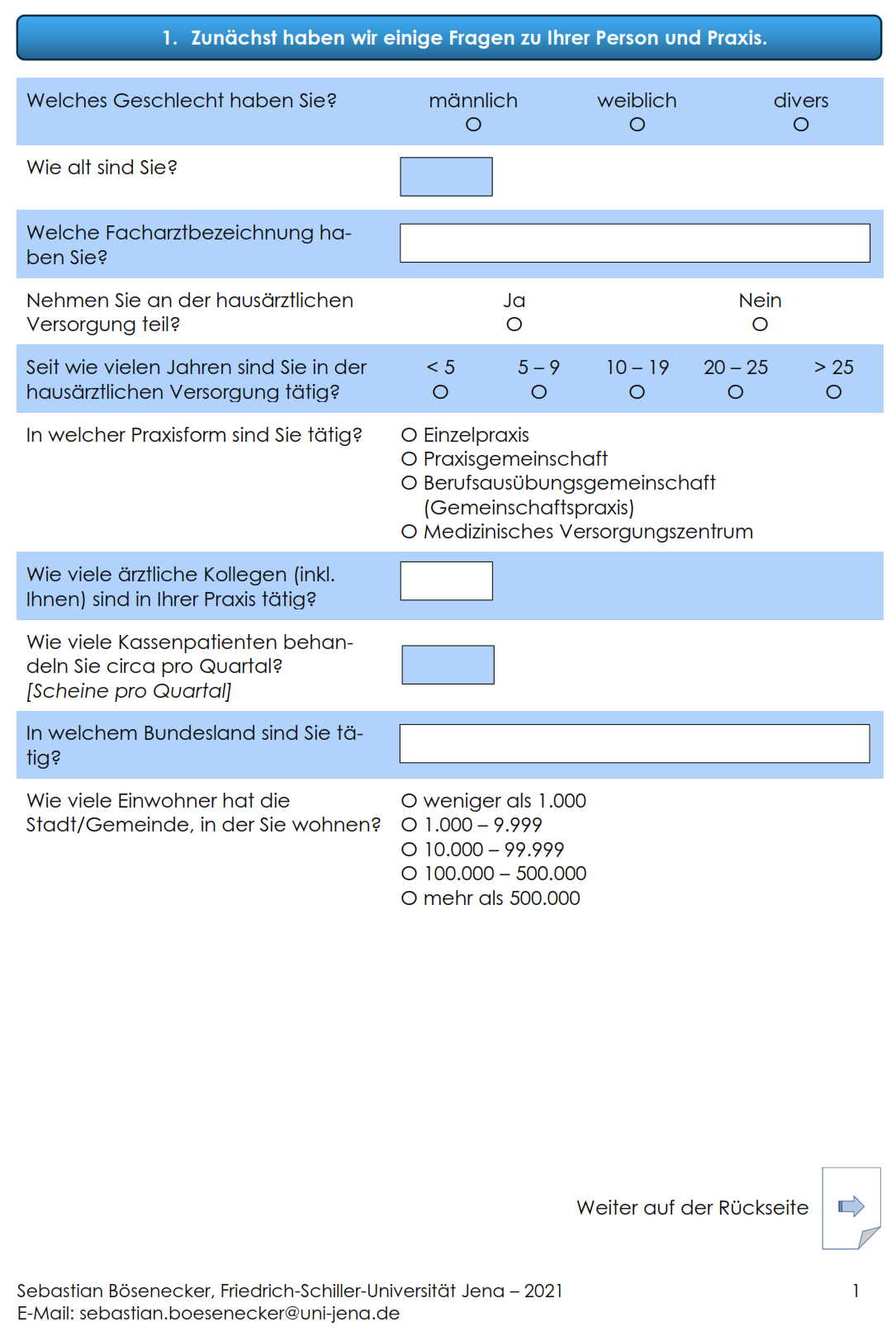

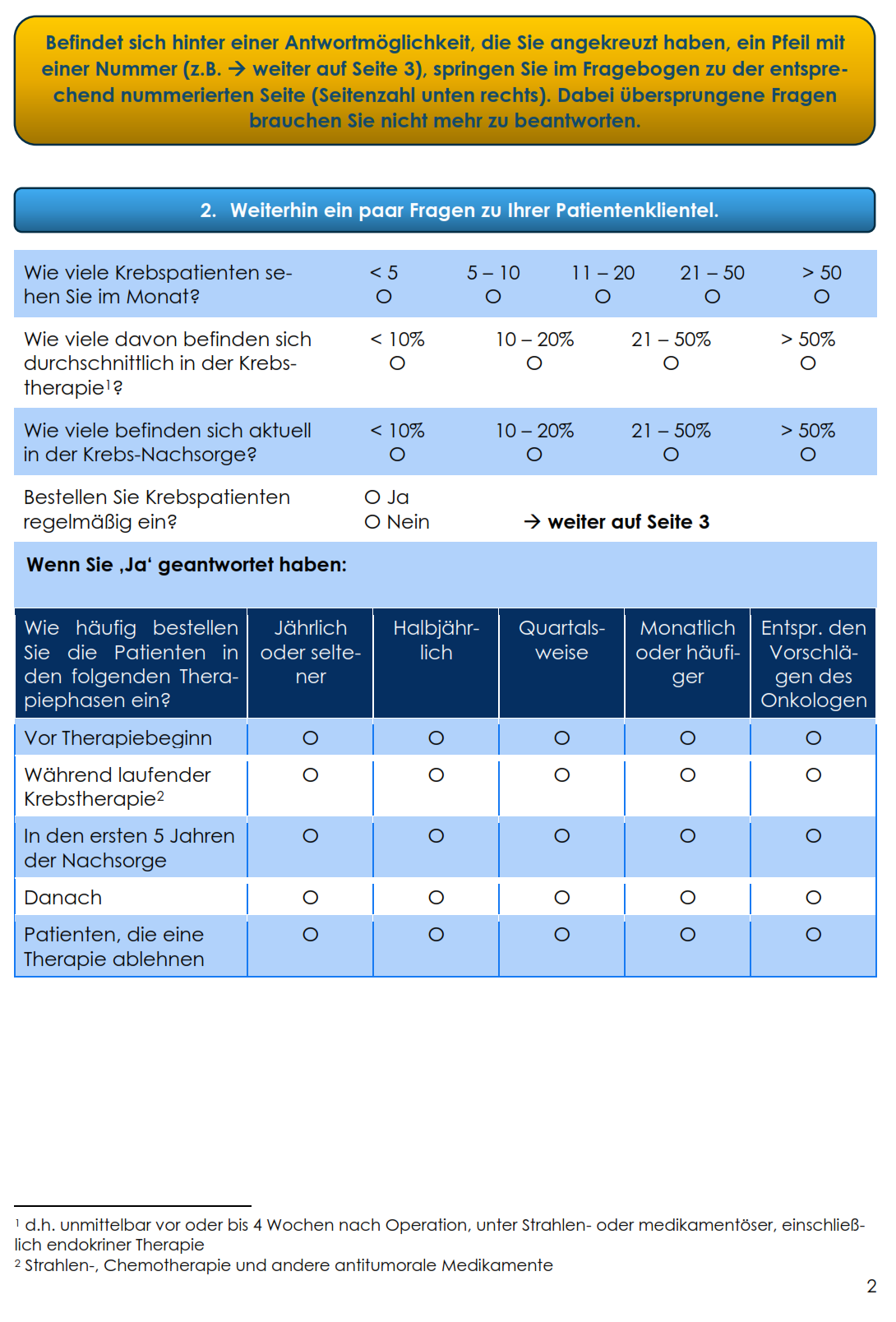

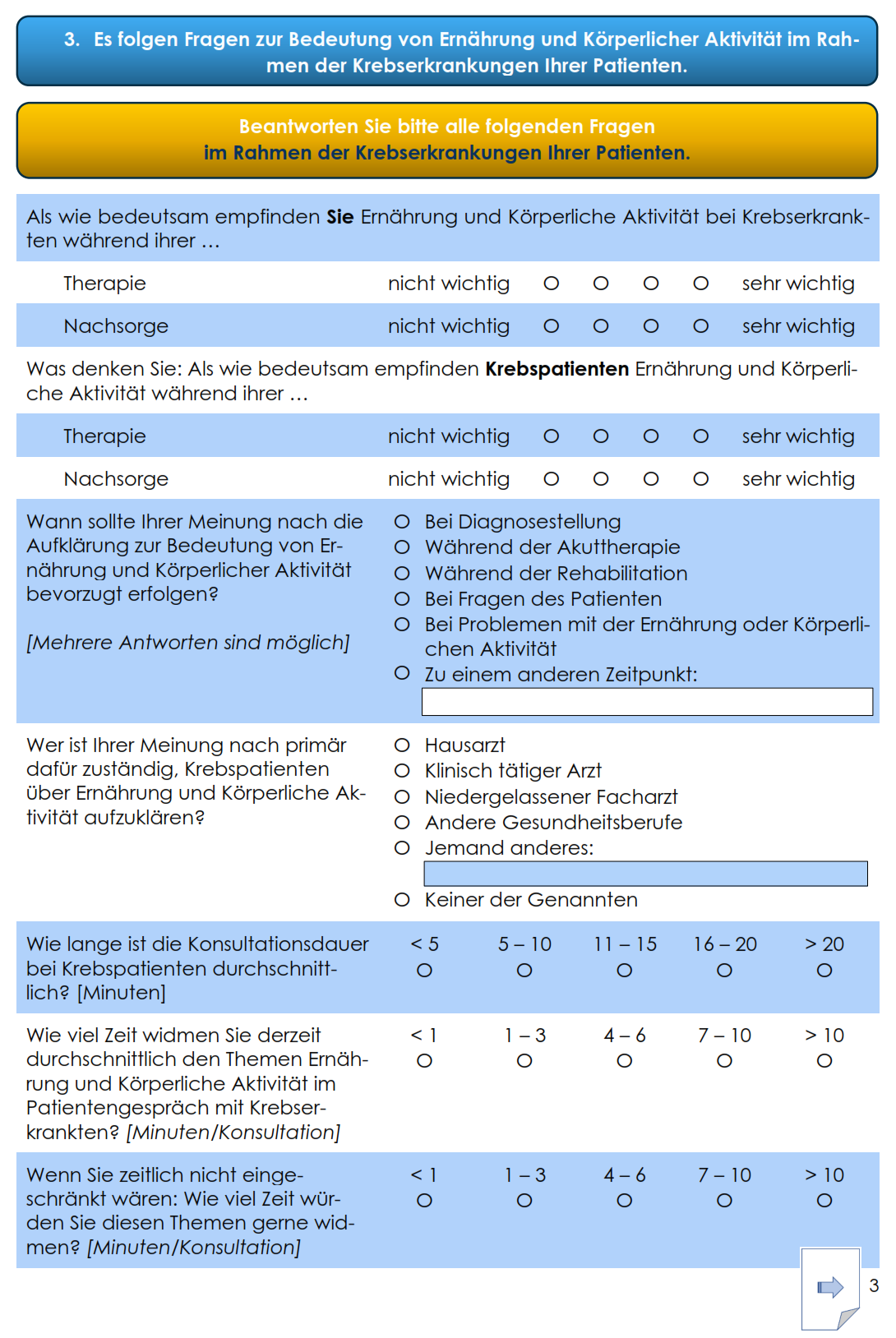

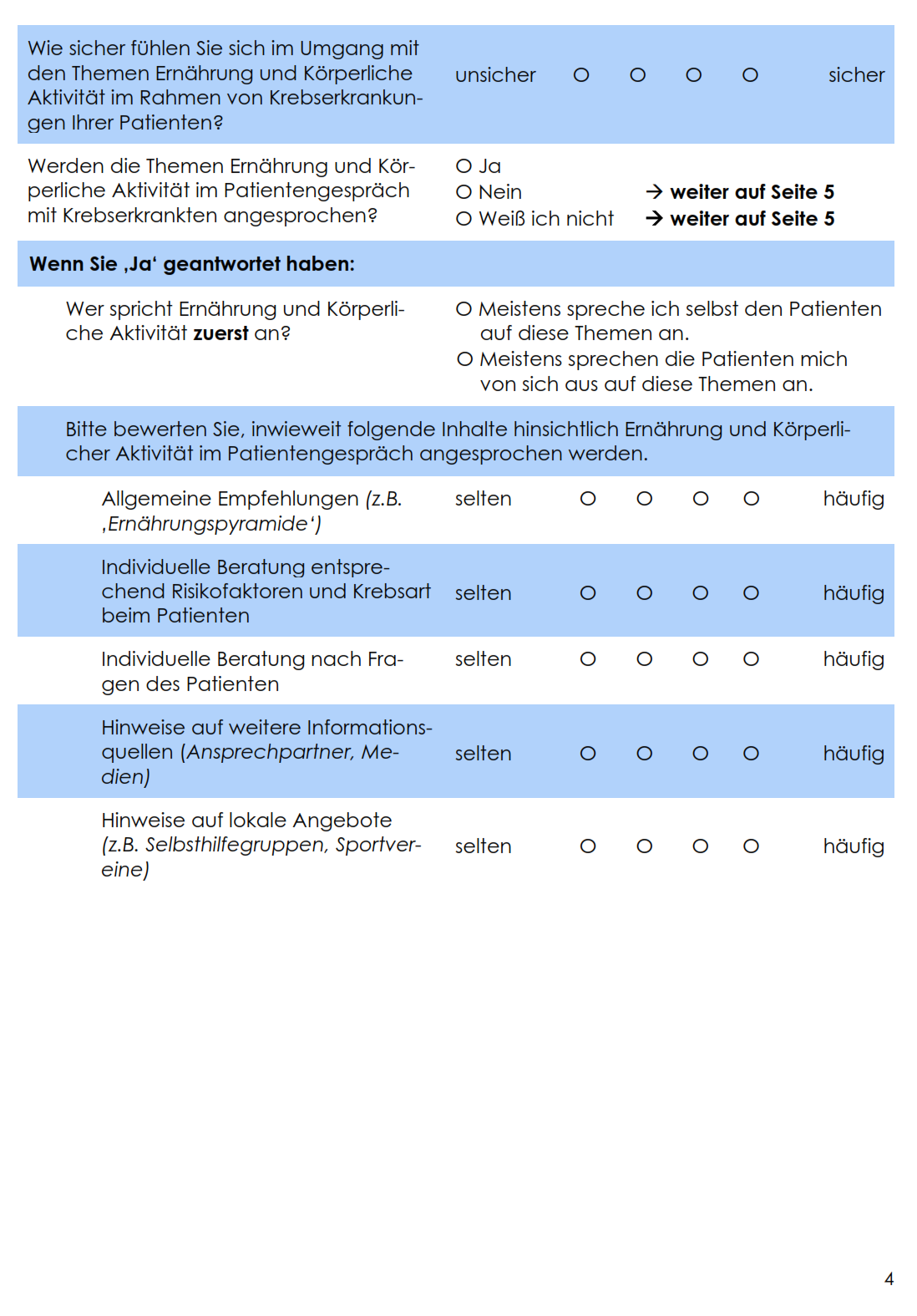

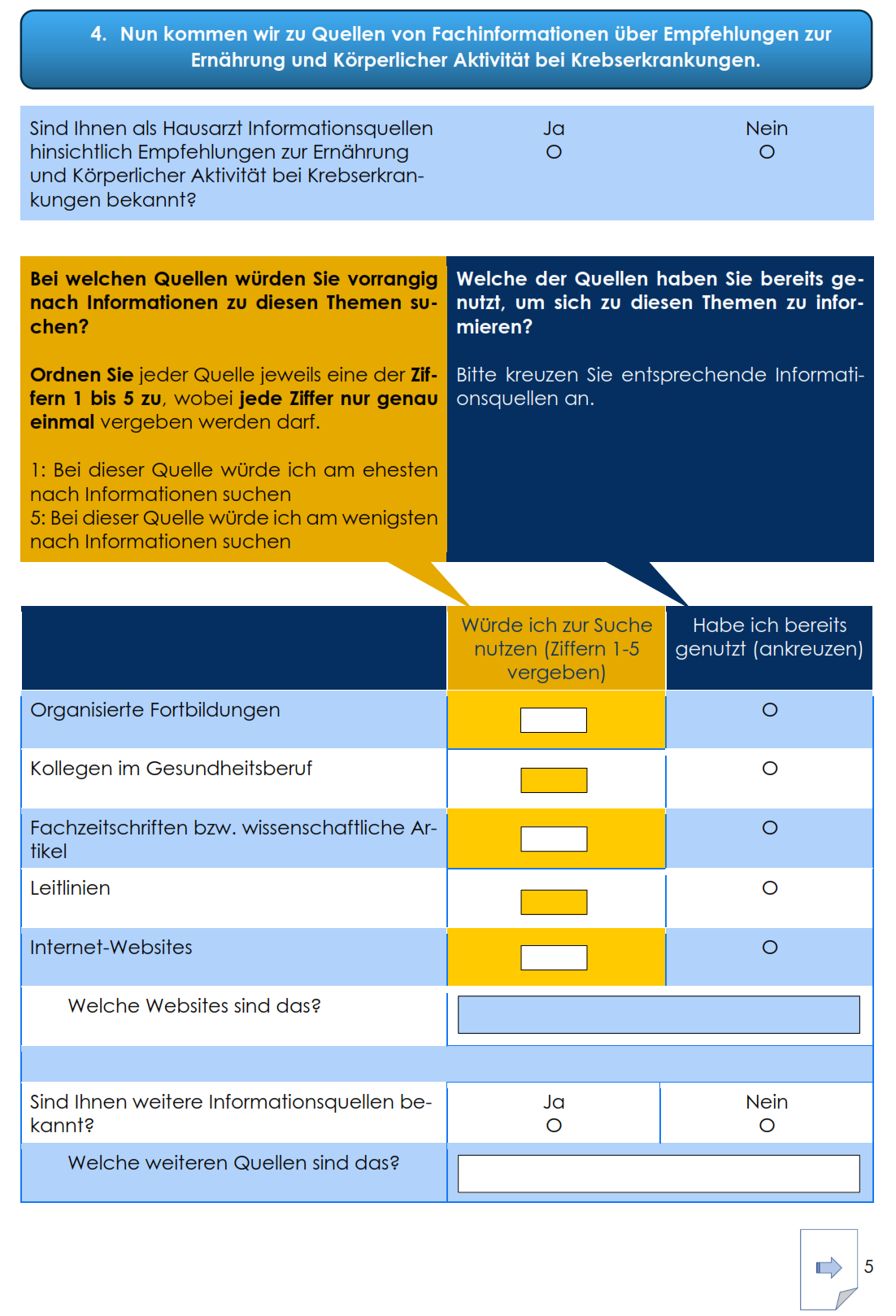

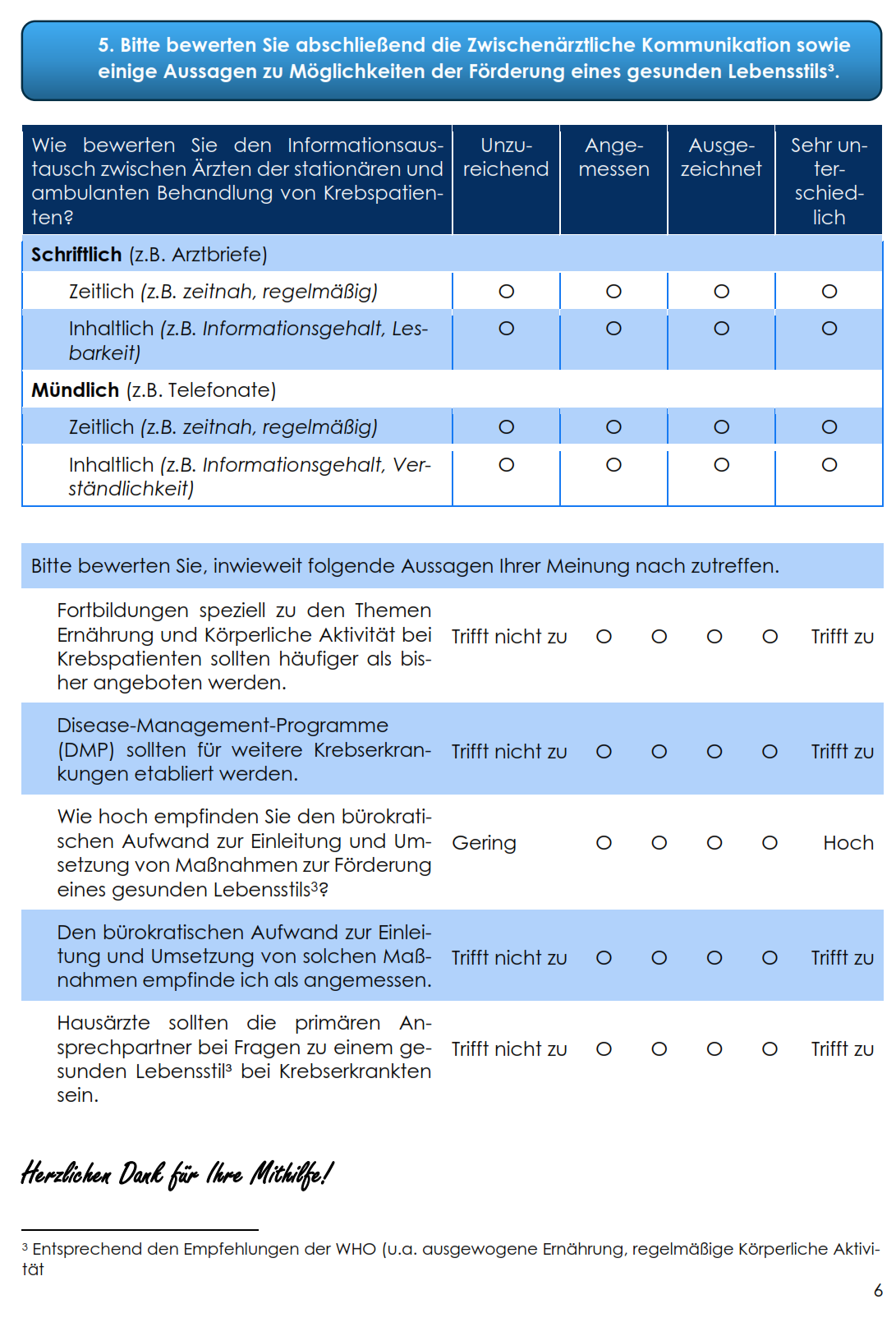

Supplement: Supplementary file 1 — Supplementary file1 (DOCX 2550 KB) [file 432_2022_4233_MOESM1_ESM.docx]
